# Dentistry in Paris

**Published:** 1881-06

**Authors:** 


### ARTICLE VIII.

### *Dentistry in Paris.*

PARIS, April 15th, 1881.

I suppose that ninety-nine out of one hundred American dentists regard Paris as a perfect El Dorado in dentistry.

There is a kind of halo hanging around any place that the mind fondly dwells upon, the farther away it is seen, and Paris is not an exception.

Æsthetically the most magnificent capital in the world, it attracts to it the cream of all nations, who, whether for a longer or a shorter stay, spend their money royally. No luxury that pampered civilization craves but will be found here in its most exquisite form, and with devotees innumerable. As dentistry is one of the luxuries of civilization (and, I might add, one of the most expensive ones too,) no wonder that the far-away dentist looks on the field with longing eyes; and when Munchausen tales are told him of the success of American dentists in Europe, what wonder that he broods over the picture in contrast with the little one horse town and its surroundings which now contain him? And in how many instances have such repinings led to an effort to sell out and "go abroad?"

Paris is full of such—not always the same, but always a full crop, the majority ripened with failure and disgust, dropping out of sight, their places to be immediately filled with new and green seed, which in turn will ripen, and be harvested like their predecessors.

Nevertheless, there are a few notable instances of success, in both professional standing and pecuniary results.

To Evans is unquestionably due much of the credit for the notoriety to which American dentistry has attained in the French capital; and that was quite as much due to his fortunate association with the second empire as to any marked superiority of professional attainments. Certainly it would be unjust to say that he conferred any greater benefits upon his *clientelle* than any one of his half a dozen later rivals. Out of thirty or more American dentists now in Paris, less than half a dozen are entitled to the distinction of being at the front; and even this ratio is probably much larger than in your more highly-favored city of New York.

The latest American experiment seems to be the four-cornered syndicate of Bogue, Moffat, Daball and Cook, in the Boulevard Hausman.

I call it an experiment, because it doth not yet quite appear that they have come to stay. It has somewhat the look of an amusement, whereby once in two years each member of the syndicate may get off for six months, and sow his wild oats, and then return to his Puritan mutton. Among those who are here to stay, Kingsley has no superior in skill or professional standing. Ten years in Paris has solved the doubts and fears of success, and established a permanent reputation.

His partner, Dr. Crane, may also be regarded as one of the highly favored ones, especially in view of the terrible ordeal which he passed a year since, and out of which he came triumphantly. That which at one time seemed disastrous beyond recovery, and threatened to drive him from Paris, took the opposite turn, and he is now more popular with his *clientelle* and the American colony than ever.

The limits of this letter will not permit justice to be done to all the other American practitioners whose skill and reputation may not be second to those mentioned.

To those ambitious young spirits ( or old ones either, as for that matter) in America who are looking with longing eyes to Paris as their ultimate professional resting place, let me say, "*don't?*" It's well enough to come here for a visit and drop your spare cash; but when that is done you will be far happier to return to the place where you earned it, and get some more.

There is hardly any place in America where it is not easier for one to *earn* a livelihood. Paris is the place to spend money; America is the place to make it.—*Herald of Dentistry.*

---

